# Differential effects of natural palm oil, chemically- and enzymatically-modified palm oil on weight gain, blood lipid metabolites and fat deposition in a pediatric pig model

**DOI:** 10.1186/1475-2891-10-53

**Published:** 2011-05-18

**Authors:** Eric N Ponnampalam, Paul Lewandowski, Kalanithi Nesaratnam, Frank R Dunshea, Harsharn Gill

**Affiliations:** 1 AgriFood Production Systems, FFSR, Department of Primary Industries, 600 Sneydes Road, Werribee VIC 3030, Australia; 2 Molecular Nutrition Unit, School of Medicine, Deakin University, Geelong VIC 3217, Australia; 3 Malaysian Palm Oil Board, 43000 Kajang, Selangor, Malaysia; 4 Melbourne School of Land and Environment, The University of Melbourne, Parkville, Vic. 3010, Australia

## Abstract

**Background:**

Increasing prevalence of obesity and overweight in the Western world, continue to be a major health threat and is responsible for increased health care costs. Dietary intervention studies show a strong positive association between saturated fat intake and the development of obesity and cardiovascular disease. This study investigated the effect of positional distribution of palmitic acid (Sn-1, 2 & 3) of palm oil on cardiovascular health and development of obesity, using weaner pigs as a model for young children.

**Methods:**

Male and female weaner piglets were randomly allocated to 4 dietary treatment groups: 1) pork lard (LRD); 2) natural palm olein (NPO); 3) chemically inter-esterified PO (CPO) and 4) enzymatically inter-esterified PO (EnPO) as the fat source. Diets were formulated with 11% lard or with palm olein in order to provide 31% of digestible energy from fat in the diet and were balanced for cholesterol, protein and energy across treatments.

**Results:**

From 8 weeks onwards, pigs on EnPO diet gained (P < 0.05) more weight than all other groups. Feed conversion efficiency (feed to gain) over the 12 week experimental period did not vary between treatment groups. Plasma LDL-C content and LDL-C/HDL-C ratio in pigs fed natural PO tended to be lower compared to all other diets. The natural PO lowered (P < 0.02) the plasma triglyceride (TG) content relative to the lard or EnPO diets, but was not different from the CPO diet. The natural PO diet was associated with lower (P < 0.05) saturated fat levels in subcutaneous adipose tissue than the CPO and EnPO diets that had lower saturated fat levels than the lard diet. Female pigs had lower lean and higher fat and fat:lean ratio in the body compared with male pigs. No difference in weight gain or blood lipid parameters was observed between sexes.

**Conclusions:**

The observations on plasma TG, muscle and adipose tissue saturated fatty acid contents and back fat (subcutaneous) thickness suggest that natural palm oil may reduce deposition of body fat. In addition, dietary supplementation with natural palm oil containing palmitic acid at different positions in meat producing animals may lead to the production of meat and meat products with lower saturated fats. An increase in fat content and a decrease in lean content in female pigs resulted in an increased body fat:lean ratio but gender had no effect on blood lipid parameters or insulin concentrations.

## Background

Increasing prevalence of obesity and overweight in the Western world, continue to be a major health threat and is responsible for increased health care costs [1,2]. High blood cholesterol, particularly low density lipoprotein cholesterol (LDL-C), high blood triglycerides (TG) and high blood glucose are major risk factors leading to the development of diseases such as cardiovascular disease (CVD), obesity and diabetes. Dietary intervention studies indicate a strong positive association between saturated fat intake and the development of obesity and CVD [3-5]. Due to this reason, there has been recommendation to replace saturated fats with unsaturated fats [6-8].

Among saturated fats, myristic (14:0), palmitic (16:0) and lauric (12:0) has been reported to be detrimental to health compared with the shorter chain (4:0 - 10:0) saturated fats [3] and unsaturated fatty acid such as oleic acid [9]. There is a considerable body of published work on the type of fatty acids (polyunsaturated vs monounsaturated vs saturated fats) and their association with disease development or prevention. Research shows that the fatty acid composition of dietary fats present in TGs mainly affects the development of obesity, diabetes and hyperlipidemia [5,10-12]. The structure of TG has been reported to be a factor affecting digestion and nutritional behaviour of lipids [4,13,14]. To date, little research has focused on understanding how the position of saturated fats within the TG molecule affects cholesterol metabolism and fat accretion.

Changes in risk factors or impairment in metabolic function due to dietary habits in early life may lead to serious metabolic disorders later in life. For example, high intake of saturated fats in early childhood may lead to changes in lipid metabolism that promotes the development of atherosclerosis in adulthood [15]. The digestion of fats by gastric and pancreatic lipases hydrolyses fatty acids from Sn-1 & 3 positions of TGs and generates free fatty acids and 2-monoglycerides [16]. Studies have shown that the absorption of palmitic acid is lower when a greater proportion of dietary palmitic acid occurs at Sn-1 & -3 than the Sn-2 position [13]. Palm olein (PO), the liquid fraction of palm oil, contains ~70% oleic acid (18:1) and 12% palmitic acid (16:0) occurring at Sn-2, i.e., most of the palmitic acid occurs at Sn-1 & -3 positions. As a result, the level of palmitic acid in the circulation of people consuming palm oil would be expected to be lower, which may result in lower blood cholesterol and tissue TG levels. However, this effect remains to be demonstrated experimentally. This study investigated the effect of positional distribution of palmitic acid in palm oil on blood lipids and fat deposition in the development of obesity, using young pigs as a model for children.

## Materials and methods

Forty weaner piglets (Large White X Landrace; 20 males and 20 females) weighing 6.38 ± 0.1 kg (Mean ± SE) were randomly allocated to one of four dietary treatments: 1) pork lard; 2) natural palm olein (NPO); 3) chemically inter-esterified PO (CPO); 4) enzymatically inter-esterified PO (EnPO) as the fat source (Table 1). The chemical modification provided altered levels of oleic acid at Sn-2, whereas enzymatic modification provided altered levels of palmitic acid at Sn-1 & Sn-3 positions [17]. Diets were formulated with 11% of lard or with palm oil (palm olein) in order to provide 16.0 MJ/kg digestible energy, 13.4% total fat and 31% of digestible energy from fat. Diets were balanced for cholesterol, protein and energy across treatments (Table 1). Following one week of adaptation on a commercial weaner ration, feed intake and body weights were recorded at 14 day intervals. Fasting blood samples were collected on days 0 (before commencement), 28, 56 & 84. Two replicates were conducted seven weeks apart. The study protocol was approved by the Animal Ethics Committee of the Department of Primary Industries, Victorian Government.

**Table 1:** Composition of diets fed lard, natural palm oil (NPO), chemically modified palm oil (CPO) and enzymatically modified palm oil (EnPO) treatment groups

Ingredient	Lard	NPO	CPO	EnPO
Wheat	42.06	42.06	42.06	42.06
Barley	15.00	15.00	15.00	15.00
Soybean meal	18.18	18.18	18.18	18.18
Meat meal	10.00	10.00	10.00	10.00
Blood meal	2.00	2.00	2.00	2.00
Dicalcium phosphate	0.54	0.54	0.54	0.54
Limestone	0.53	0.53	0.53	0.53
Salt	0.20	0.20	0.20	0.20
Vitamin-Mineral Premix	0.20	0.20	0.20	0.20
Lysine	0.14	0.14	0.14	0.14
Threonine	0.15	0.15	0.15	0.15
Tylan	0.08	0.08	0.08	0.08
Test Oil	-	11.0	11.0	11.0
Lard	11.0	-	-	-
Cholesterol	-	0.1	0.1	0.1

Values are expressed as g/100 g diet (%) on air dry basis.

### Sample collection

At the end of feeding period, animals were slaughtered humanely at a commercial abattoir. Back fat depth (P2) was measured at 12^th ^rib, 65 mm away from the midline (spinal cord), which is a standard measure to assess the carcass fat content. Carcass fatness was assessed after the removal of visceral components and some pelvic and kidney fat tissues if present in excess level as a standard for commercial procedure. The left side of each carcass was transported by a refrigerated truck to the Meat Research and Training Centre, Werribee, Victoria for sampling of muscle and adipose tissue and measurement of body composition. Muscle (50 g) and fat (20 g) tissues were collected from *longissimus lumborum *site (i.e., loin muscle area above 13^th ^rib). Half carcasses (left sides) were weighed and the composition of each half carcass was determined using a Hologic QDR4500 dual energy X-ray absorptiometry (DXA) machine, using a calibration equation [18]. Weights of lean tissue, fat tissue, bone mineral content and total carcass weight were calculated for each carcass using the calibration equation and converted to percentages of total carcass weight as described in Ponnampalam et al. [19].

### Plasma lipids

Blood was transported to the laboratory on ice packs within 2 hours of collection and then centrifuged at 3000 g for 10 minutes. Plasma was then collected into aliquots and frozen at -80°C for further analysis of total cholesterol, high-density lipoprotein cholesterol (HDL-C), low-density lipoprotein cholesterol (LDL-C), triglycerides (TG), insulin and glucose. Total plasma cholesterol, HDL-C, and plasma TGs were determined via the use of colorimetric kits (Thermo Electron, Sydney, Australia) in accordance with the manufacturer's instructions. Low density lipoprotein cholesterol (LDL-C) was determined using the Friedewald equation [20]: LDL-C = Total Cholesterol - HDL-C - (TG/5). Plasma insulin was measured using a porcine insulin RIA Kit (Linco Research, St. Charles, MO) in accordance with the manufacturer's instructions.

### Fatty acid determination in muscle and adipose tissue

Lipid extractions and fatty acid analyses of meat samples were carried out in duplicate. Seven gram portions of minced homogenized muscle or 0.5 gram of fat (subcutaneous adipose) tissue were extracted with 60 mL of chloroform-methanol (2:1 v/v) containing 10 mg/L of butylated hydroxytoluene and 5 mg of methyl tricosanoate as internal standard (C23:0, Nu-Chek-Prep, Ely-sian, MN, USA). Following extraction overnight and filtering, 8 mL aliquot of the filtrate was mixed with 2 mL of 0.9% NaCl, shaken and left overnight at 4°C to remove aqueous impurities. On the following day, the lower phase containing lipids was evaporated with pure nitrogen gas and fatty acids methyl esters (FAME) of the total lipids were prepared by the addition of 1 mL of toluene and 3 mL of 0.9 M H_2_SO_4 _in methanol and heating the resulting solution at 70°C for 2h with shaking at 15 min intervals.

Upon cooling, 3 mL of petroleum ether and 3 mL of distilled water was added. This mixture was then thoroughly mixed and centrifuged for 10 min at 1000 rpm. The fatty acid containing upper phase was separated in a screw-capped tube, evaporated to dryness and reconstituted with petroleum ether. The fatty acid methyl esters were separated by capillary gas liquid chromatography using a 60 m × 0.32 mm fused silica bonded phase column (BPX70, SGE, Melbourne, Australia). Fatty acids were identified by comparison with standard mixtures of FAME (Nu-Chek-Prep, Elysian, MN, USA), and the results were calculated using response factors derived from chromatograph standards of known composition as determined previously [21].

### Statistical analysis

Data were subjected to Analysis of Variance (ANOVA) using Genstat version 10.1. Data collected from all 40 animals on feed intake, growth, weight gain, FCR, carcass fatness and blood lipid profiles were analysed for the main effect of dietary treatments and sexes. The means of treatment groups and sexes for blood lipids and insulin were analysed using ANOVA with initial concentrations, at day 0, as covariates. For the determination of carcass composition as fat, lean (muscle) and bone mineral (ash) percentages or weights (mass), carcass weight was used as a covariate. Means were compared using least significant differences (LSD) and P < 0.05 was considered statistically significant.

## Results

### Feed intake, growth, weight gain and gain/Kg feed consumption of animals

**Figure 1:**
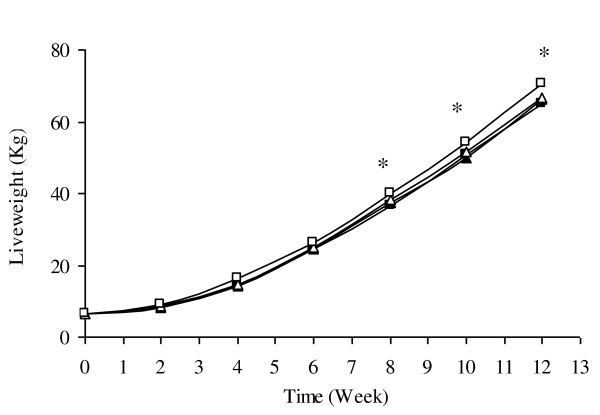
Mean live weight of animals over the 12 week feeding period. Data is presented as mean across all treatment for 12 weeks of feeding period. *Indicates that EnPO (opened square) gained significantly more weight from 8 weeks onwards compared with lard (closed black square), NPO (closed black triangle) and CPO (opened triangle) groups (P < 0.05).

Animals on EnPO diet consumed 14-15 kg more feed (P < 0.05. Table 2) over the 12 week period compared with other treatments. As a consequence, there was a greater increase in body weight (P < 0.001; Table 2) and weight gain (P < 0.001; Table 2) in EnPO animals after the 12 weeks of feeding compared with lard, NPO and CPO. Weight gain per kilogram feed consumed (FCR) was similar between all treatments, ranging from 0.49-0.51 kg gain/kg of feed consumed (Table 2). Weekly body weight records further indicated that animals consuming diets supplemented with EnPO gained significantly more weights from 8 weeks onwards than the other groups (P < 0.05, Figure 1). There were no differences between sexes in feed intake, weight gain and gain per kg feed intake (Table 2).

**Table 2:** Feed intake, final liveweight, weight gain, gain per feed, carcass weight, back fat depth and carcass composition as weights (mass) or percentages (%) of pigs after 12 weeks of feeding lard, natural palm oil (NPO), chemically modified palm oil (CPO) and enzymatically modified palm oil (EnPO) diets*

	Lard	NPO	CPO	EnPO	Female	Male	SEM Diet	SEM Sex
Feed intake (Kg)	116.4^a^	117.2^a^	117.3^a^	131.1^b^	120.3	120.7	4.46	2.65
Final liveweight	65.0^a^	66.3^a^	66.9^a^	70.4^b^	66.2	68.1	1.75	3.23
Weight gain (Kg)	58.6^a^	59.9^a^	60.5^a^	64.1^b^	59.7	61.9	1.72	2.93
Gain/feed	0.50	0.51	0.51	0.50	0.50	0.52	0.02	0.02
Carcass weight (kg)	52.5	52.5	53.2	53.5	52.7	53.1	1.46	0.97
Back fat depth (mm)	13.2^b^	11.2^a^	12.6^ab^	12.3^ab^	12.09	12.56	0.82	0.90
Half carcass fat (kg)	4.92	4.78	4.95	4.77	5.09^x^	4.62^y^	0.31	0.15
Half carcass lean (kg)	17.59	17.74	17.53	17.63	17.39^x^	17.86^y^	0.29	0.14
Half carcass bone (ash, kg)	0.70	0.69	0.69	0.70	0.70	70	0.01	0.01
Fat (%)	21.2	20.5	21.5	20.8	21.9^x^	19.9^y^	0.93	0.76
Lean (%)	75.9	76.6	75.6	76.3	75.1^x^	77.1^y^	0.86	0.62
Ash (%)	3.03	3.02	3.03	3.02	3.02	3.03	0.03	0.01
Fat:lean ratio	0.28	0.27	0.29	0.27	0.29^x^	0.26^y^	0.02	0.01

^*^Means are average of 10 observations for treatments and 20 observations for sexes. Data were presented as mean ± SEM.

^a,b,c & x,y^Means with different superscripts between dietary treatments and sexes, respectively differ significantly at P < 0.05.

### Carcass fatness

Body fatness evaluated by back fat measurement at the P2 site (a measure of external adipose tissue thickness) was lower (P < 0.05) in NPO animals compared with lard fed animals, but CPO and EnPO were not different from NPO or lard treatments (Table 2). Carcass composition assessed by DXA showed no differences in carcass percentages (fatness, muscle content and bone content) or by weights (mass) between treatment groups (Table 2). Females had lower body lean content (P < 0.01), higher fat content (P < 0.02) and higher fat:lean ratio (P < 0.02) than male pigs.

### Blood lipid profiles

There was no difference in total cholesterol, LDL-C or HDL-C or insulin levels between treatments after 12 weeks of feeding (Table 3). Blood TG levels were lower in animals fed NPO diet compared to lard and EnPO (P < 0.02, Table 3). There were no differences in the concentrations of cholesterol, TG, LDL-C, HDL-C or insulin between males and females (Table 3).

**Table 3:** Blood total cholesterol, LDL-cholesterol (LDL-C), HDL-cholesterol (HDL-C), ratio of LDL-C/HDL-C, triglycerides (TG) and plasma insulin concentrations of pigs at week 12 of feeding lard, natural palm oil (NPO), chemically modified palm oil (CPO) and enzymatically modified palm oil (EnPO) diets*

	Lard	NPO	CPO	EnPO	Female	Male	SEM Diet	SEM Sex
Cholesterol (mmol/L)	3.38	3.37	3.57	3.56	3.90	3.04	0.77	0.74
LDL-C (mmol/L)	1.87	1.69	2.00	2.04	2.25	1.55	0.64	0.50
HDL-C (mmol/L)	1.51	1.68	1.57	1.52	1.59	1.55	0.19	0.22
LDL-C:HDL-C ratio	1.21	1.17	1.38	1.44	1.58	1.03	0.29	0.44
Triglycerides (mmol/L)	0.49^b^	0.30^a^	0.39^ab^	0.64^b^	0.41	0.50	0.11	0.14
Insulin (μU/ml)	3.40	3.09	4.15	4.57	3.28	4.33	1.18	0.91

Initial concentrations were used as covariates for the analysis.

^*^Means are average of 10 observations for treatments and 20 observations for sexes. Data presented as mean ± SEM.

^a,b,c^Means with different superscripts between dietary treatments differ significantly at P < 0.05.

### Muscle and adipose tissue fatty acid composition

Myristic acid (14:0) and stearic acid (18:0) content in muscle from all palm oil fed pigs was significantly lower than lard fed pigs (P < 0.01, Table 4). Similarly, muscle palmitoleic acid (16:1) content was also significantly lower in all palm oil fed pigs compared to lard fed pigs (P < 0.01, Table 4), with NPO and CPO fed pigs displaying lower palmitoleic acid levels than EnPO fed pigs. Adipose tissue total saturated fatty acid content from all palm oil fed pigs was significantly lower than lard fed pigs (P < 0.05, Table 5), with NPO fed pigs displaying lower total saturated fatty acid levels than CPO and EnPO fed pigs. Similarly, adipose tissue myristic acid (14:0) content was also significantly lower in all palm oil fed pigs compared to lard fed pigs (P < 0.03, Table 5), with NPO and CPO fed pigs displaying lower myristic acid levels than EnPO fed pigs.

**Table 4:** Fatty acid composition (%) of muscle longissimus of pigs after 12 weeks of feeding lard, natural palm oil (NPO), chemically modified palm oil (CPO) and enzymatically modified palm oil (EnPO) diets*

	Lard	NPO	CPO	EnPO	Female	Male	SEM Diet	SEM Sex
12:0	0.06	0.06	0.06	0.06	0.06	0.06	0.004	0.002
14:0	1.12^b^	0.95^a^	0.92^a^	0.96^a^	1.1^x^	0.95^y^	0.06	0.028
16:0	24.5	24.9	25.3	25.0	25.2^x^	24.7^y^	0.41	0.21
16:1	2.79^b^	2.35^a^	2.26^a^	2.47^ab^	2.59^x^	2.35^y^	0.17	0.09
18:0	11.6^b^	10.5^a^	10.9^a^	10.5^a^	10.9	10.8	0.30	0.27
18:1	43.2	44.5	42.3	43.7	44.1^x^	42.7^y^	1.51	0.53
18:2	14.5	14.7	16.1	15.2	14.1^x^	16.2^y^	1.38	0.70
Total SFA	37.9	36.9	37.7	37.1	37.8	37.1	0.67	0.44
Total MUFA	46.0	46.9	44.5	46.1	46.7^x^	45.1^y^	1.61	0.52
Total PUFA	16.1	16.2	17.7	16.7	15.6^x^	17.8^y^	1.51	0.74

^*^Means are average of 10 observations for treatments and 20 observations for sexes. Data presented as mean ± SEM.

^a,b,c & x,y^Means with different superscripts between dietary treatments and sexes, respectively differ significantly at P < 0.05.

Abbreviations: SFA, saturated fatty acids; MUFA, monounsaturated fatty acids; PUFA, polyunsaturated fatty acids.

**Table 5:** Fatty acid composition (%) of subcutaneous fat (adipose) tissue of pigs after 12 weeks of feeding lard, natural palm oil (NPO), chemically modified palm oil (CPO) and enzymatically modified palm oil (EnPO) diets*

	Lard	NPO	CPO	EnPO	Female	Male	SEM Diet	SEM Sex
12:0	0.07	0.07	0.11	0.08	0.11	0.07	0.07	0.04
14:0	1.53^b^	1.10^a^	1.06^a^	1.23^ab^	1.20	1.26	0.16	0.14
16:0	27.3^b^	22.5^a^	24.8^ab^	26.5^ab^	25.3	25.3	2.74	2.62
16:1	1.29	1.14	0.94	1.07	1.11	1.10	0.24	0.23
18:0	11.6^b^	8.75^a^	9.58^ab^	9.98^ab^	9.71	10.3	1.36	0.90
18:1	39.9^a^	50.2^b^	48.4^ab^	44.5^ab^	46.2	45.3	4.84	3.91
18:2	16.2	15.1	13.9	15.3	14.8	15.4	1.65	1.52
18:3	0.75	0.59	0.48	0.54	0.63	0.56	0.13	0.11
Total SFA	41.4^b^	32.7^a^	35.9^ab^	38.3^ab^	36.8	37.3	3.30	2.47
Total MUFA	41.2^a^	51.3^b^	49.4^ab^	45.6^ab^	47.4	46.4	4.75	3.95
Total PUFA	17.4	15.9	14.6	16.1	15.9	16.3	1.76	1.63

^*^Means are average of 10 observations for treatments and 20 observations for sexes. Data presented as mean ± SEM.

^a,b,c^Means with different superscripts between dietary treatments differ significantly at P < 0.05.

Abbreviations: SFA, saturated fatty acids; MUFA, monounsaturated fatty acids; PUFA, polyunsaturated fatty acids.

In the muscles, females had greater (P < 0.05) levels of 14:0, 16:0, 18:1 than male pigs. Total PUFA was higher (P < 0.02) in males while total monounsaturated fats were higher (P < 0.02) in females (Table 4). In adipose tissues, there were no differences in fatty acids between males and females (Table 5). There was no significant diet x sex interactions for feed consumption, weight gain, blood or muscle and adipose tissues.

## Discussion

### Performance of animals and fatness in the body

Feed intake data shows that pigs on EnPO diet gained significantly more weight than all other groups. However this did not influence the feed to gain ratio (feed conversion efficiency), fat deposition or fat:lean ratio in the body over the whole experimental period. Body fatness evaluated by back fat measures indicated that the NPO diet significantly lowered subcutaneous adipose tissue deposition (external fat deposition) compared with the lard diet. These observations are consistent with the findings of [22] who reported similar increases in feed intake and weight gain. In contrast however, these researchers found increases in back fat thickness rather than a reduction. However there was no effect of other palm oil diets (CPO & EnPO) compared to NPO or lard diets. The reduction in adipose tissue thickness with NPO was not evident from carcass fatness, as determined by DXA, although NPO fed animals had a slightly lower percent fat than the other treatment groups. Muscle and bone mineral contents (%) determined by DXA showed similar levels between dietary groups. Although feed consumption and weight gain were not affected by sex, females had greater fatness and fat:lean ratio compared to males, suggesting that dietary energy was diverted to fat deposition in females. This could be due to fundamental differences between males and females in the development of lean and fat mass in the body. Young females (18-20 years) have been reported to have significantly higher fat body mass by weight and % fat body mass compared with their male counterparts, which is similar to the findings in the present study [23].

### Plasma metabolites and fatty acid content in muscle and adipose tissue

Heart disease is one of the leading causes of mortality in the Western world. Due to the link between obesity and heart disease, prevention of heart disease through dietary means has focused almost exclusively on reducing intake of cholesterol and fat. Triglycerides are the compounds that help fat move through the bloodstream. People who have high levels of LDL-C and low levels HDL-C often have high TGs [24,25]. Elevated TG levels are increasingly associated with increased risk for heart disease [25,26]. The present data show that natural palm oil is beneficial compared to CPO or EnPO or lard in terms of lowering blood TGs that are associated with obesity and heart disease. After 12 weeks feeding lard or palm oil diets, the total cholesterol and LDL-C levels in blood did not change. Plasma LDL-C content and the ratio of LDL-C to HDL-C of pigs fed natural PO tended to be lower compared to all other diets (Table 3). Because of the younger age of experimental animals, blood cholesterol, LDL-C, HDL-C and insulin did not differ significantly.

The results showed a lower saturated fatty acid deposition in tissues of muscle (14:0 & 18:0; P < 0.001; Table 4) and subcutaneous fat (14:0, 16:0, 18:0 & SFA; P < 0.05; Table 5) with palm oil diet. This is consistent with our findings of a reduction in subcutaneous fat thickness with NPO diet compared to lard. The hydrolysis of the triglyceride components of chylomicrons and very low density lipoproteins in the circulatory systems is catalysed by lipoprotein lipase enzyme (LPL). The LPL enzyme is mainly located on the capillary surface within muscle and adipose tissues for hydrolysis of circulating lipoproteins, thereby providing free fatty acids and 2-monoglycerides for tissue utilisation i.e., either for the deposition of fatty acids in tissues or energy release through mitochondrial oxidation of free fatty acids. The lower saturated fat levels (P < 0.05) in subcutaneous adipose tissue with NPO diet could be due to lower levels of circulating TG (Table 3) which may influence fatty acid deposition in the body. The lower saturated fat levels in subcutaneous adipose tissue with NPO had also resulted in a smaller (P < 0.05) adipose tissue thickness (P2 fat) in those animals. This effect might have been due to a cumulative process of decrease in saturated fat deposition such as palmitic and stearic acids with natural palm oil feeding. Because the melting point of saturated fats is higher than monounsaturated fats, they provide structure and insulation to the external body through their firmness. The data on plasma TG, tissue fatty acids and back fat (subcutaneous) thickness indicates that natural palm oil may have beneficial effects on obesity through reduction in fatness in the body [27]. The reduction in saturated fats in the body needs further investigation. There was evidence of sex difference; females had higher saturated (14:0, 16:0) and monounsaturated (16:1, 18:1) fats in the muscles than males while the polyunsaturated (18:2n-6) fat was higher in males than females. Adipose tissue fatty acid composition did not change between sexes.

The results also show that the significant reduction found in back fat depth with NPO diet was in line with the reduction in saturated fat content in adipose tissue (back fat depth), plasma LDL-C and TG levels. Since we have used younger pigs (weaners at 4 weeks age) in the study, the greater proportion of dietary energy would have been utilised for muscle and bone growth while the energy utilised for fat deposition would be expected to be lower. However, we hypothesised that consumption of all diets with high saturated fat in early life may not lead to the development of obesity and cardiovascular diseases later in life. The findings of this study support the suggestion that positional distribution of palmitic acid present in TG structure of palm oil confers a favourable plasma lipid profile and fat deposition in the body, which may prevent the development of obesity.

Together, these results suggest that feeding NPO to adult pigs may elicit even more healthful effects in terms of cholesterol synthesis and lipid metabolism. This is because, a greater proportion of the dietary metabolisable energy would be utilised for fat deposition as a result of decline in protein synthesis and bone growth with ageing or maturity, in adult humans/adult pigs. In pigs [28] and sheep [29] maintained under same feeding background, carcass fatness and leanness (muscle) obtained at different stages indicated that as the age increased, lean muscle content decreased and fat content increased in the body. In addition, application of diets with natural palm oil containing palmitic acid at different positions to meat producing animals may lead to the production of meat and meat products with lower saturated fats.

## Conclusions

Natural PO diet significantly lowered the plasma TG content relative to the lard or EnPO diets, but was not different from the CPO diet. Plasma LDL-C content and the ratio of LDL-C to HDL-C in pigs fed NPO tended to be lower compared to all other diets. The lower saturated fat content in subcutaneous adipose tissue with NPO diet could be due to lower levels of circulatory TG that was observed in that group compared with lard diet. This effect appears to be related to palm oil's positional distribution of palmitic acid in the TG moiety. An increase in body fat content and a decrease in lean content in female pigs resulted an increased fat:lean ratio, but sex had no effect on blood lipid parameters or insulin concentration. In general, the lipid metabolism and the fat deposition in adult pigs are different from young pigs. This is because in young animals the metabolisable energy drawn from diet is mainly used for muscle/bone growth and development. In adult pigs, while the muscle accretion declines with age, the major portion of dietary energy is diverted to fat deposition. Feeding palm oil to adult pigs may provide more favourable outcomes in terms of lowering blood TG levels and fat deposition in the body. However, this needs to be investigated in adult pigs including the whole body composition analysis using dual energy X-ray absorptiometry (DXA).

## Competing interests

The authors declare that they have no competing interests.

## Authors' contributions

ENP carried out the experimental study as a principle investigator and drafted the manuscript. PL carried out the analysis of blood for lipid metabolites and insulin concentrations, and participated in the interpretation of data. ENP, FRD, KN, and HG participated in the project proposal development, design of the study, the statistical analysis, and interpretation of data. Technical staff from the Department of Primary Industries conducted the tissue fatty acid analysis under ENP's supervision. All authors read and approved the final manuscript.
